# Supplementary diet components of little auk chicks in two contrasting regions on the West Spitsbergen coast

**DOI:** 10.1007/s00300-014-1568-9

**Published:** 2014-09-26

**Authors:** Rafał Boehnke, Marta Gluchowska, Katarzyna Wojczulanis-Jakubas, Dariusz Jakubas, Nina J. Karnovsky, Wojciech Walkusz, Slawomir Kwasniewski, Katarzyna Błachowiak-Samołyk

**Affiliations:** 1 Institute of Oceanology Polish Academy of Sciences, Powstańców Warszawy 55, 81-712 Sopot, Poland; 2 Department of Vertebrate Ecology and Zoology, University of Gdańsk, Wita Stwosza 59, 80-308 Gdańsk, Poland; 3 Department of Biology, Pomona College, 175 W. 6th St., Claremont, CA 91711 USA; 4 Freshwater Institute, Fisheries and Oceans Canada, 501 University Crescent, Winnipeg, MB R3T 2N6 Canada

**Keywords:** Little auk, Feeding ecology, Arctic, Zooplankton, Svalbard, Climate change

## Abstract

The complete diet composition structure of the most numerous planktivorous sea bird, little auk (*Alle alle*), in the European Arctic, is still not fully recognized. Although regular constituents of little auk chick diets, the copepods, *Calanus glacialis* and *C. finmarchicus* have been previously relatively well described, more taxa were frequent ingredients of the bird’s meals. Therefore, the role of the little auks supplementary diet components (SDCs) at two colonies in the Svalbard Archipelago, Hornsund and Magdalenefjorden, in 2007–2009, is a main subject of this comparative study. Because the SDCs often consisted of scarce but large zooplankters, this investigation was focused on biomass as a proxy of the SDCs’ energy input. Although the total biomass of the food delivered to chicks in both colonies was similar, in Magdalenefjorden, the proportion of SDCs was twice that found in Hornsund. The main SDCs in Hornsund were Decapoda larvae (with predominating *Pagurus pubescens*) and *Thysanoessa inermis*, whereas the main SDCs in Magdalenefjorden were *C. hyperboreus* and *Apherusa glacialis.* Previous investigations, which indicated lipid richness of SDCs, together with our ecological results from the colonies, suggest that this category might play a compensatory role in little auk chick diets. The ability to forage on diverse taxa may help the birds to adapt to ongoing Arctic ecosystem changes.

## Introduction

The little auk (*Alle alle*) is one of the most numerous seabirds of the northern Atlantic (Stempniewicz 2001), breeding exclusively in the High Arctic (Isaksen and Gavrilo 2000). It is a planktivorous bird with metabolic rates considered as one of the highest among seabirds (Gabrielsen et al. 1991; Konarzewski et al. 1993). Due to extremely high energy demands, little auks rely almost exclusively on lipid-rich zooplankton associated with cold Arctic waters (Kwasniewski et al. 2012). Since foraging little auks prefer Arctic water masses, avoiding the Atlantic ones with smaller and less profitable zooplankton community, this species is considered as an important indicator of changes in the marine environment (Karnovsky et al. 2003; Jakubas et al. 2007; Stempniewicz et al. 2007; Harding et al. 2008). For that reason, the breeding ecology of the little auk, including aspects of feeding strategy and chick diet composition, has been frequently investigated in the context of an ongoing climate-induced changes in the Arctic (e.g., Jakubas et al. 2007; Harding et al. 2009; Fort et al. 2010; Karnovsky et al. 2010; Kwasniewski et al. 2010; Jakubas et al. 2011; Kwasniewski et al. 2012).

Previous studies have shown that copepods are the most important components in the diet of little auk chicks raised in the large colonies on Svalbard, i.e., Bjørnøya (Węsławski et al. 1999), Isfjorden (Steen et al. 2007), Hornsund (Karnovsky et al. 2003; Wojczulanis et al. 2006; Jakubas et al. 2007; Kwasniewski et al. 2010; Jakubas et al. 2011; Kwasniewski et al. 2012) and Magdalenefjorden (Kwasniewski et al. 2010; Jakubas et al. 2011; Kwasniewski et al. 2012). In particular, *Calanus glacialis*, a highly caloric calanoid copepod associated with cold Arctic water, was found to be the most important energy source for birds from Svalbard colonies. *Calanus finmarchicus* is of secondary importance. This copepod has lower energy content and is associated with warm Atlantic waters (e.g., Kwasniewski et al. 2010 and citations therein). Although many other zooplankters such as amphipods, euphausiids, decapods, ostracods, gastropods, cephalopods, and polychaetes have been reported to occur in the diet of the little auk (e.g., Wesławski et al. 1999; Karnovsky et al. 2003; Wojczulanis et al. 2006; Steen et al. 2007), the role of such supplementary diet components (hereafter SDCs) has never been a subject of a separate, detailed investigation. The purpose of this study was to assess and compare the role of the SDCs in chick diets of little auks from the years 2007–2009 in the two breeding colonies with contrasting oceanographic conditions in order to provide a more complete understanding of the bird’s feeding biology.

## Materials and methods

### Study area

Data on diet composition were collected in 2007–2009 at two large little auk colonies located on the west coast of Spitsbergen in Hornsund (Ariekammen slope, 77°00′N, 15°31′E) and Magdalenefjorden (Høystakken and Alkekongen slopes 79°35′N, 11°05′E).

The Hornsund area is influenced by two currents; the coastal Sørkapp current providing cold and less saline Arctic water and the West Spitsbergen Current (WSC) transporting warmer, more saline Atlantic water (Piechura et al. 2001; Cottier et al. 2005; Piechura and Walczowski 2009). In the Magdalenefjorden area, the relatively warm and saline water of the WSC is present over the large part of the shelf, while fresher and colder Arctic type water travels as an anticyclonic coastal current, visible as a narrow flow close to the coast (Kwasniewski et al. 2010; Jakubas et al. 2011).

### Collection of materials

Food loads were taken from adult birds transporting food to their offspring throughout the breeding season. In total, 173 diet food samples were collected in Hornsund (55, 55, and 63) and 170 in Magdalenefjorden (41, 70, and 59) in 2007, 2008, and 2009, respectively. The birds were captured randomly in the colony by mist nets or noose carpets and the contents of their gular pouch were gently scooped out with a spoon. Each diet sample was put in a separate plastic bag and preserved in 4 % borax-buffered formaldehyde. General diet results from Hornsund and Magdalenefjorden from 2007 to 2009 were already presented in Kwasniewski et al. (2010, 2012), Fort et al. (2010), Harding et al. (2011), Jakubas et al. (2011), Kidawa et al. (2014), Hovinen et al. (2014), and Karnovsky et al. (2010).

### Laboratory processing and data analyses

**Fig. 1:**
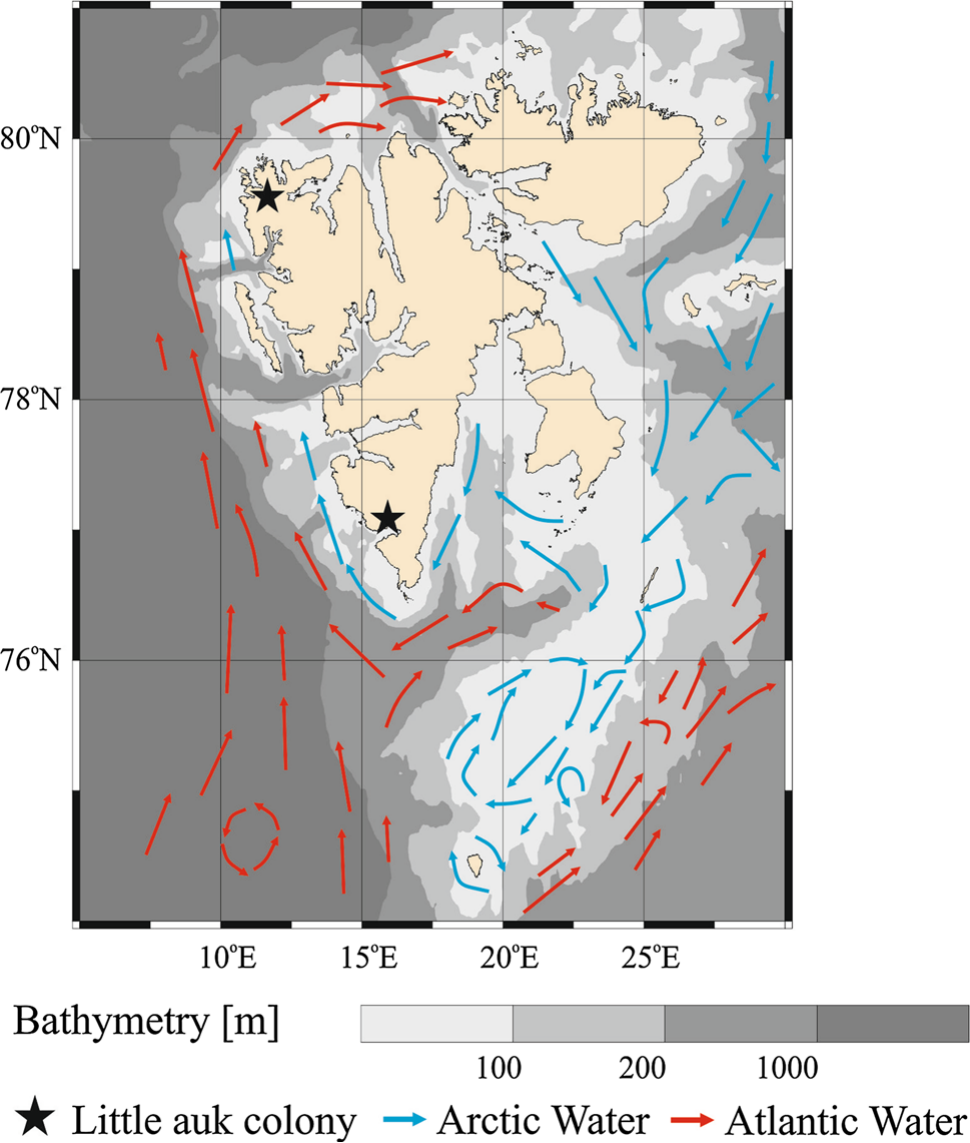
Study area in the Spitsbergen region with sea current pattern (after Loeng and Drinkwater 2007, modified), bathymetry (depth contours and gray shading), the locations of little auk colonies (*black stars*)

All samples collected from the adult little auks in the colonies were subjected to quantitative and qualitative laboratory analyses following the procedures described in Kwasniewski et al. (2010). Due to a lack of inter-annual differences, data from the 3 years of observations were pooled to allow comparisons between the two colonies. “Supplementary diet components” (SDCs) were defined as all zooplankters found in little auk chick diets, except for *C. glacialis* and *C. finmarchicus,* which are known to be the two main components of their diet (Karnovsky et al. 2003, 2010; Kwasniewski et al. 2010). Because the importance of SDCs is related to the amount of energy they can provide, we used their dry mass instead of abundance to investigate their role and compare it between the colonies. To compare the chick meals from different colonies, χ^2^ test for independence was conducted. The proportions of particular items in diet components (Hornsund colony vs. Magdalenefjorden colony), as well as the proportions of the selected SDCs in the total SDCs biomass between colonies (Hornsund vs. Magdalenefjorden), were compared for yearly pooled samples using χ^2^ test for independence (2 × 2 table). The total biomass of chick meals, as well as the biomass of SDCs in the chick meals, were compared using Mann–Whitney *U* test. To compare the frequency distribution of SDCs contribution to the chick meals, biomass between the colonies, nonparametric Kolmogorov–Smirnov two-samples test was used. Statistical analyses were done using STATISTICA 10.0 (StatSoft, Inc.) following recommendation by Sokal and Rolf (1981), and the significance level for all tests used was *p* < 0.05 (Fig. 1).

## Results

### Taxonomic composition of little auk chick diet

A total of 36 zooplankton taxa were recorded in the diet of little auk chicks during the whole study period in the two colonies combined. Of that, nine taxa made up 99 % of the total diet biomass. Two main diet components: *Calanus. glacialis* and *C. finmarchicus* were present in 96 and 95 % of the diet samples, respectively (data not shown). Seven other taxa most frequently observed in diet samples were as follows: *C. hyperboreus* (52 % of all diet samples)*, Apherusa glacialis* (17 %)*, Thysanoessa inermis* (23 %)*, Decapoda* larvae (excluding *Pagurus pubescens* larvae, 51 %), *Pagurus pubescens* larvae (59 %), *Themisto libellula* (36 %), and *T. abyssorum* (73 %). The remaining 27 taxa (*Thysanoessa raschii* and *T. longicaudata*, fish larvae, *Paraeuchaeta* spp., *Hyperoche medusarum, Hyas araneus* larvae, *Metridia longa*, *Limacina helicina*, *Gammarus wilkitzkii*, and *Eukrohnia hamata,* together made up 1 % of the diet biomass and were therefore combined into one group of the SDCs, the “remaining category”.

### Biomass composition of chick diet

**Fig. 2:**
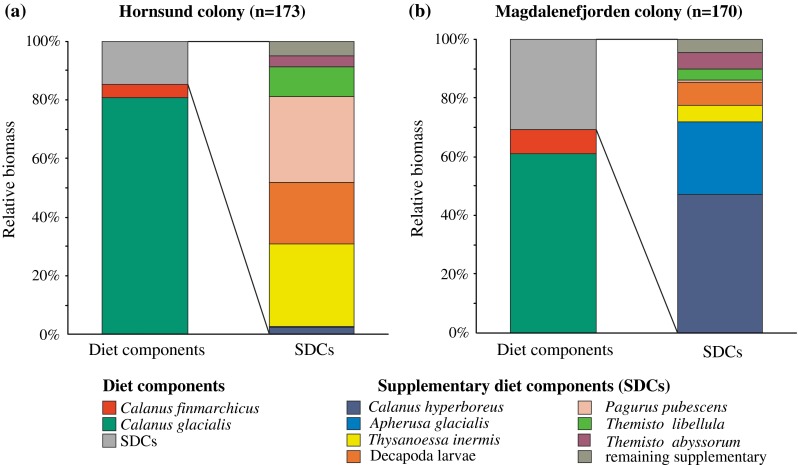
Relative composition of zooplankton prey biomass in the food delivered to little auk chicks in **a** Hornsund and **b** Magdalenefjorden colonies in 2007–2009, combined, with details of the biomass composition of birds supplementary diet components (SDCs)

Dry mass (DM) of the food delivered to chicks was similar in Hornsund (min = 21.5 mg, max = 2,535.2 mg; Me = 902.7 mg DM meal^−1^ *n* = 173) and Magdalenefjorden (min = 83.1 mg, max = 2,177.8 mg; Me = 925.3 mg DM meal^−1^ *n* = 170; Mann–Whitney *U* test, Z_173,170_ = −0.83, *p* = 0.406). Similarly, no differences were found in the mean biomass of the SDCs between Hornsund (59.7 mg DM meal^−1^) and Magdalenefjorden (40.7 mg DM meal^−1^; Z_173,170_ = −1.69, *p* = 0.09). The food delivered to chicks by parent birds in the Hornsund colony differed from the food delivered by the birds in Magdalenefjorden with regard to biomass contributions of prey items (test χ^2^, χ^2^ = 9.71 *df* = 2, *p* = 0.008: Fig. 2a, b). In Magdalenefjorden, the chick meals had a lower proportion of *C. glacialis* (χ^2^ test, χ_1_^2^ = 14,820.1, *p* < 0.001), a higher proportion of *C. finmarchicus* (χ_1_^2^ = 1,919.8, *p* < 0.001) and the proportion of SDCs twice of that found in Hornsund colony (χ^2^ test, χ_1_^2^ = 11,331.8, *p* < 0.001).

### Contribution of SDCs in chick diet

The contributions of different prey items to the SDCs biomass differed significantly between the two studied colonies (test χ^2^, χ^2^ = 110.9 *df* = 7, *p* < 0.001: Fig. 2a, b). The main components of SDCs in Hornsund were: *Pagurus pubescens* larvae (29 % of SDCs biomass)*, Thysanoessa inermis* (28 %), Decapoda larvae (21 %), amphipods *Themisto libellula* and *T. abyssorum* (14 %), as well as *C. hyperboreus* (3 %) (Fig. 2a). In Magdalenefjorden, the predominant SDCs taxa were *C. hyperboreus* (47 %), *Apherusa glacialis* (25 %), Decapoda larvae (8 %), *T. inermis* (6 %), *T. abyssorum* and *T. libellula* (10 %), and *Pagurus pubescens* larvae (1 %) (Fig. 2b). The proportions of each of the above-mentioned food items to the total SDCs biomass differed between the colonies (χ^2^ tests, *p* < 0.001, individual test values not shown).

### Intercolony differences in the contribution of SDCs in chick diet

**Fig. 3:**
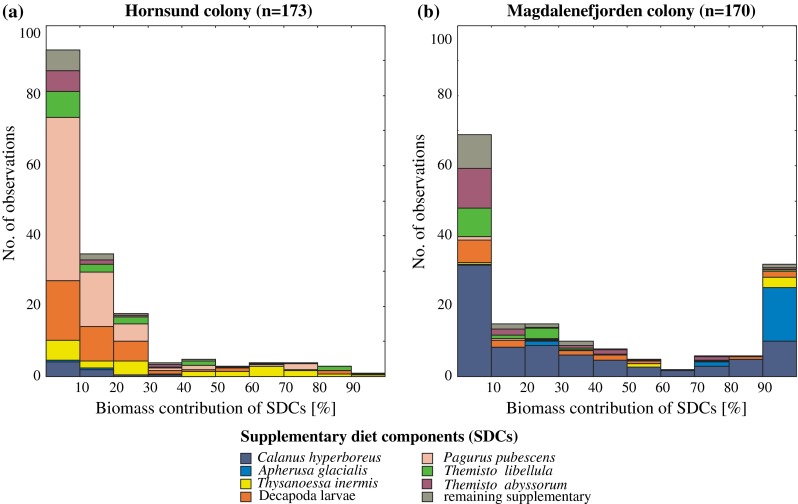
Frequency distribution of contributions to biomass of supplementary diet components (SDCs) for Hornsund (**a**) and Magdalenefjorden (**b**)

Frequency distribution of SDCs contribution to the total chick meals biomass was different between the colonies (Kolmogorov–Smirnov test, *p* < 0.001; Fig. 3a, b). In the diet samples collected in Hornsund colony, the SDCs most frequently made up <10 % of the diet, and 84 % of the samples had the SDCs contribution lower or equal to 30 %. In Magdalenefjorden, the most frequently found class of SDCs contribution was also <10 %, but only 58 % of the samples had up to 30 % of the SDCs. Only in 1 % of the samples from Hornsund and almost in 20 % from Magdalenefjorden the SDCs contribution was larger than 90 %.

Among the samples with SDCs contribution <30 %, the most important food items in Hornsund were *Pagurus pubescens* and Decapoda larvae (Fig. 3a), whereas in Magdalenefjorden the main item was *Calanus hyperboreus* (Fig. 3b). Among the samples with SDCs contribution >30 %, the most important food items in Hornsund were euphausiids (predominantly *T. inermis*) while in Magdalenefjorden they were *A. glacialis* and *C. hyperboreus*. What is more, in Magdalenefjorden, *A. glacialis* alone made up the bulk of the biomass in the samples with >90 % contribution of the SDCs to the little auk chick diet.

## Discussion

An overall diversity of taxa found in little auk chick diet in the two study colonies (36 taxa) was similar to that reported previously from Hornsund (35 taxa; Karnovsky et al. 2003), but was higher than in Isfjorden, western coast of Spitsbergen (23 taxa; Steen et al. 2007), and substantially higher than in northwest Greenland (14 taxa; Pedersen and Falk 2001), BjØrnØya (18 taxa; Węsławskiet al. 1999) and Franz Josef Land Archipelago (13 taxa; Węsławski et al. 1994). Greater diversity of taxa in the diet found in our study compared to some other colonies may reflect more intense sampling effort. This, in turn, may be attributed to more complex oceanographic conditions in the vicinity of the two studied colonies. Presence of the two greatly different water masses (influenced by Arctic and Atlantic currents) results in co-occurrence of two distinct zooplankton assemblages—Arctic and boreal—of which each contains own zooplankton diversity (e.g., Karnovsky et al. 2003; Kwasniewski et al. 2010).

*Calanus hyperboreus* was the dominant SDC of little auk chicks’ meals in the Magdalenefjorden colony with 47 % share in the SDCs biomass (15 % of total diet biomass). In a study from East Greenland, this copepod was the dominant component of chick diets with biomass consisting of 36–58 % of the total diet (Fort et al. 2010; Karnovsky et al. 2010). The prevalence of the last-mentioned component was also observed in northwest Greenland (Frandsen et al. 2014). This taxon exhibits better-developed lipid storage strategy, and is much larger than its two counterparts, *C. finmarchicus* and *C*. *glacialis* (Hagen and Auel 2001). Adult *C. hyperboreus* inhabits mostly deep waters, but their immature copepodid stages occupy surface waters (Hirche et al. 2006), therefore they can serve as an available and very nourishing food source for diving little auks.

The ice-associated amphipod, *Apherusa glacialis*, was the second most important SDC in the little auk chicks’ food in Magdalenefjorden. This amphipod was present in 20 % of the samples from that colony. In fact, 9 % of the food loads consisted exclusively of *A. glacialis*. Our study confirmed the previous finding that little auks breeding in Magdalenefjorden forage regularly in the marginal sea ice zone, located even up to 110 km from the colony (Jakubas et al. 2012, 2013), where this ice-associated amphipod can be found (Jakubas et al. 2011). *Apherusa glacialis* helps meet the high energy demands of little auks due to its high lipid content (Scott et al. 1999). The low share of *A. glacialis* in the little auk diet in Hornsund (Fort et al. 2010) was also confirmed in our study. Higher abundance of *A. glacialis* was found in little auk food loads in Hornsund in 2004 when the sea ice was observed in the adjacent foraging area (Jakubas et al. 2007). The higher importance (32 % of all prey items biomass) of ice-associated fauna in the diet of little auks from East Greenland (Fort et al. 2010) is most likely related to the proximity of the sea ice habitat in this area. The situation of the birds from the colony in Magdalenefjorden (this study), where 8 % of the total diet components biomass (comprising 25 % of the SDCs biomass) was made up by the ice associated prey item, suggests that birds from the colony could forage in the ice-edge area.

Amphipods of *Themisto* genus were also noticeable SDCs in the food loads of the little auk chicks (14 % in Hornsund and 10 % in Magdalenefjorden). Similarly, *Themisto* equaled to 17 % of SDCs biomass have been reported in the little auk diet at Hakluyt Island (northwestern Greenland, Pedersen and Falk 2001). In the present study, the contribution of *T. libellula* was higher in Hornsund (10 % of SDCs biomass) (Fig. 2a), while *T. abyssorum* in Magdalenefjorden (6 %) (Fig. 2b). Due to the relatively high amount of lipids, mainly wax esters (Auel et al. 2002), amphipods from genus *Themisto* play an important role in the diet of different seabirds like the Brunnich’s guillemot (*Uria lomvia*) and black-legged kittiwake (*Rissa tridactyla*) (Lønne and Gabrielsen 1992).

In Hornsund, *Pagurus pubescens* was the predominant SDC, with 29 % share in SDCs biomass. In the previous studies in Hornsund, this prey item made up 2 and 8 % of the total chicks diet biomass (Karnovsky et al. 2003 and Fort et al. 2010, respectively). During our investigation, it constituted only 4 and 1 % of the total chick diet biomass in Hornsund and Magdalenefjorden, respectively. Although *P. pubescens* is relatively low caloric (~3 kcal g ^−1^ DW; according to Wacasey and Atkinson 1987), it seemed to be attractive prey for little auks likely because of its high availability near the Hornsund colony (Boehnke, unpubl.).

The euphausiid *Thysanoessa inermis*, found in the chick diets in our study, is more tolerant to colder water than its shallow-water and less-energetic counterpart *T. raschii* (Falk-Petersen et al. 1982; Hagen and Auel 2001). *Thysanoessa inermis* is associated with Arctic waters and in our study this species was a considerable contributor to SDCs biomass (28 %) of the little auk’s diet in Hornsund. In the birds diet from Isfjorden, only a few individuals of *Thysanoessa* spp. were observed (Steen et al. 2007), but in the Franz Josef Archipelago, *T. inermis* constituted ~11 % of the total little auk’s prey biomass (Węsławski et al. 1994).

Although in over half of the diet samples SDCs constituted only up to 10 % of total chicks diet biomass, in 25 diet samples (constituting 15 % of samples) taken in Magdalenefjorden those predominant SDCs (*C. hyperboreus* and *A. glacialis*) constituted almost the whole sample (Fig. 3b).

The high frequency of occurrence of ice-associated fauna in the diet samples (our study) seems to confirm the previous assumption that little auks breeding in West Spitsbergen coast are able to modulate their feeding ecology depending on the temporal availability of their prey and also to exploit a wide range of prey (Karnovsky et al. 2008; Fort et al. 2010). The latest study of Hovinen et al. (2014) revealed that provisioning rates in little auks in two colonies in West Spitsbergen (including Magdalenefjorden) did not influence the chick’s probability to fledge. Additionally, the same investigation showed that sea surface temperature was negatively correlated with fledging probability. This was likely related to the prey availability and quality in the little auk’s foraging grounds. Thus, in our study, little auks from Magdalenefjorden most probably compensated lower availability of preferred *C. glacialis* on the adjacent foraging grounds (data not shown) by delivering other prey items, in that some energy-rich species, which was possible due to the relatively close distance to the marginal sea ice zone. This flexibility may help little auks to adapt to the expected ecosystem variability due to climate change by feeding on novel zooplankton species, which may increase in the North Atlantic as a result of global warming (Fort et al. 2010).
